# The environmental geochemical baseline, background and sources of metal and metalloids present in urban, peri-urban and rural soils in the O´Higgins region, Chile

**DOI:** 10.1007/s10653-021-01098-4

**Published:** 2021-10-09

**Authors:** Ana Valdés Durán, Guillermo Aliaga, Katja Deckart, Cyrus Karas, Dante Cáceres, Adriana Nario

**Affiliations:** 1 Centro de Tecnologías Nucleares en Ecosistemas Vulnerables, División de Investigación y Aplicaciones Nucleares, Comisión Chilena de Energía Nuclear, Las Condes, Nueva Bilbao, 12501 Santiago, Chile; 2 Fundación Añañuca, Camino La Laguna S/N, San Vicente de Taguatagua, Región del Libertador Bernardo O’Higgins, Chile; 3 Departamento de Geología, Facultad de Ciencias Físicas y Matemáticas, Universidad de Chile, Plaza Ercilla 803, Santiago, Chile; 4 Advanced Mining Technology (AMTC), Facultad de Ciencias Físicas y Matemáticas, Universidad de Chile, Tupper, 2007 Santiago, Chile; 5 Departamento de Ingeniería Geográfica, Universidad de Santiago, Av. B. O’Higgins, 3363 Santiago, Chile; 6 Programa de Salud Ambiental, Escuela de Salud Pública, Universidad de Chile, Avenida Independencia 939, Recoleta, Santiago, Chile; 7 Facultad de Ciencias de la Salud, Universidad de Tarapacá, General Velásquez 1775, Arica, Chile

**Keywords:** Geochemistry, Soils, Pollution, Metals, Metalloids, Environment

## Abstract

The importance of environmental geochemistry baseline in soils of O´Higgins Region, Chile, since it hosts in its eastern area one of the major Cu-Mo producing mines in the country, is to establish and explain relationships between the chemical compositions of the Earth’s surface and potential contaminants sources such as mining industry, agriculture and urban activity. A total of 109 samples of urban, peri-urban and rural soils were analyzed with X-ray fluorescence to determine most of the elemental concentrations analyzed. The C and S analyses were performed with the high-temperature combustion method, and a MERCUR mercury analyzer was used for Hg. The study shows that the distribution patterns for most major elements and some trace elements are controlled by the lithologic substrate. This study identified areas with metals and metalloids in high concentrations, which are a risk to the environment and health according to established international regulations. Some of these components correspond to Cu (2500 ppm), Mo (26,5 ppm), As (134,6 ppm), Cr (206.6 ppm), Hg (0.2 ppm), Ni (26.4 ppm), Pb (61.7 ppm), V (227,2 ppm) and Zn (180.3 ppm). Through an elementary association analysis, most of these elements resulted from extractive activities of Cu, metal alloys and oil combustion. It was also possible to trace the use of fertilizers and pesticides in agricultural soils, as well as the combustion of oil related to vehicles in the study area. This information is relevant to implement environmental management strategies to control possible exposure to toxic compounds to human health.

**Supplementary Information:**

The online version contains supplementary material available at 10.1007/s10653-021-01098-4.

## Introduction

During the recent decades, the increase in metals and metalloid pollution levels is probably related to anthropogenic factors such as industrial emissions, agricultural activities and vehicular emissions (Albanese et al., 2008). These increased levels can be distinguished from natural variabilities stemming from different lithologies and geogenic processes, such as erosion, weathering and transport (Albanese et al., 2008). Hence, determining a baseline is important to accurately describe variations in element concentrations in surface environments (Salminem and Tarvainen, 1997; Salminem and Gregorauskiene, 2000). These baselines are usually represented as value ranges that demonstrate the heterogeneity of the environment (Reimann et al., 2005) and allow for the determination of contaminated areas and the evaluation of anthropogenic impact on the environment and public health (Bhat et al., 2019).

Chile geodynamically has a subduction zone with an active volcanic arc located in the High Andes, which explains, in some cases, increased copper and arsenic concentrations between other metals present in the Earth’s crust. Since the beginning of last century, Chile has sustained its economy with mining activity, characterized by the presence of porphyry copper molybdenum and epithermal gold located mainly in the center and north of the country (Oyarzún et al., 2016). The total amount of tailings in this section is estimated to be about 742, classified in actives, non-actives, abandoned and some with a lack of information, according to the National Survey of Mining and Geology of Chile (SERNAGEOMIN). Most of these deposits are located within the transversed valleys oriented from east to west, where the fluvial action acts as transport mechanisms for mine waste, which is considered a potential source of pollution. This can be added to other activities such as agriculture and industry, whose emissions also have impacts on hydric resources, soils and air.

The variety of soils present in Chile is not only determined by the climatic difference from north to south and from east to west, but also from the characteristics and properties of the lithology.

In this context, environmental geochemistry through the generation of soil baselines considers the compositional information of the parent material, determining original concentrations, allowing the quantification of the current content of metals and metalloids and their variation with respect to natural concentrations (background), facilitating better control of the levels of potential pollutants. In Chile, to date, there are only active sediment baselines generated by SERNAGEOMIN. Although there are studies of soil characterization and discrimination between background and anthropic contribution, which mostly focus on local issues (Carcovick et al., 2016), made mostly in the north of Chile (Tapia et al., 2018).

The present work intends to contribute to the recognition of polluted soils. The use of metal and metalloid concentrations and the statistical treatment of the entire data set will allow the characterization of these soils and establish a baseline of the study area. Therefore, the main contribution of this study is the realization of a first baseline of soil geochemistry in urban, peri-urban and rural soils, identifying areas with potential toxic compounds corresponding to high concentrations of inorganic components that can represent a risk to the environment and its population. In addition, the traceability of the main polluting sources responsible for these geochemical anomalies is carried out.

## Materials and methods

### Study area

**Fig. 1:**
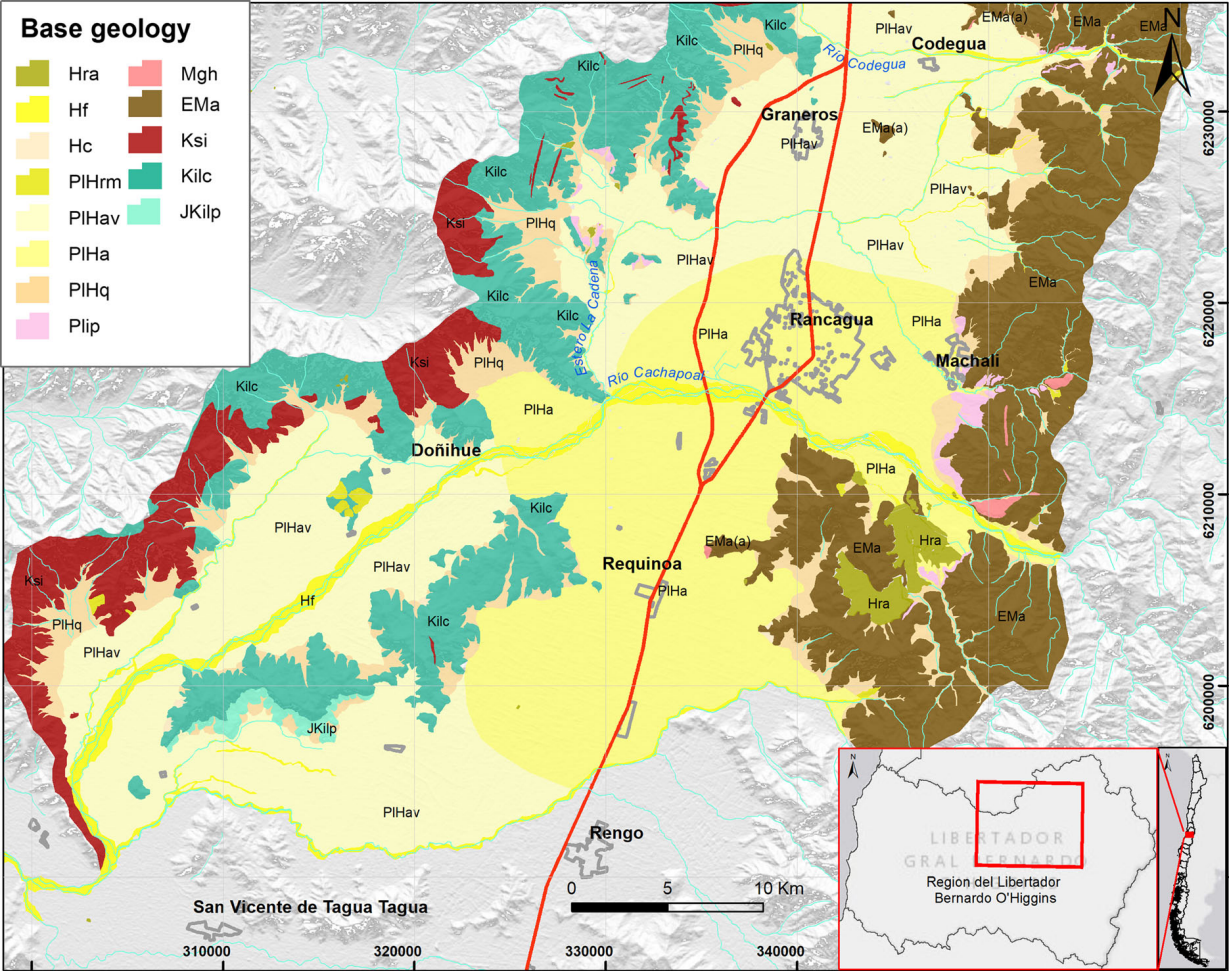
Geological map of Rancagua- San Vicente de Tagua (Alfaro et al., 2017; Godoy et al., 2009).* JKilp*: Lo Prado Formation (Titonian–Hauterivian) formed by volcano and sedimentary sequences of marine—continental origin, *EMa*: Cenozoic volcanic and sedimentary sequences, Abanico Formation; *Hf*: River deposits (Holoceno); *Hra*: anthropogenic deposits; *Kilc*: Lower Cretaceous volcanic and sedimentary sequences, Las Chilcas Formation; *Ksi*: Upper Cretaceous plutonic and hypabyssal stocks; *Mgh*: stocks and plutons, veins and manto-veins of Miocene age; *PlHa*: Alluvial deposits; *PlHac*: piedmont alluvial deposits; *PlHrm*: mass transport deposits; *Hf*: modern fluvial deposits along the current river beds; *Hra*: anthropogenic deposits

The present study consists of a soil geochemical characterization carried out in the area located between 34°S–34°15’S and 70 °30′W–71 °W of the O’Higgins region, central Chile (Fig. 1). This area includes Rancagua, the regional capital (60 km^2^), Machalí (15 km^2^), Codegua (12 km^2^), Doñihue (10 km^2^), Graneros (9 km^2^) and Requinoa (7.5 km^2^), as well as rural and inter-rural areas located between villages. The area is known for its agricultural activity, associated with the use of fertilizers and pesticides. There is also a series of pollutant sources, such as agricultural burns, vehicular traffic and welding workshops. To the east (67 km) of Rancagua at the foot of the Andes Mountains is the porphyry-type copper (Cu) deposit “El Teniente,” which is one of the biggest underground mines in Chile (Dold and Fontboté, 2001). This deposit experienced non-stop Cu and molybdenum (Mo) extraction for more than a century, producing the tailing dam deposits at Barahona 1, Barahona 2 and Laguna Cauquenes (Kelm et al., 2009). Previous studies of the soils in the same region corroborate the high concentration of some metals and metalloids in the analyzed soils and active assets (Ahumada et al., 2004; Ascar et al., 2008a, b).

### Geology of the study area

The study area is located in the Central Depression, composed by Pleistocene–Holocene sedimentary deposits (Alfaro et al., 2017; Godoy et al., 2009; Fig. 1). One of the oldest formations in this zone is the Lo Prado Formation (Titonian–Hauterivian) (*JKilp*) formed by volcano and sedimentary sequences of marine—continental origin. The valley is to the west limited by the Coastal Cordillera, which consists of Lower Cretaceous volcanic and sedimentary sequences (*Kilc*) (Las Chilcas Formation) and Cretaceous plutonic and hypabyssal stocks (*Ksi*). To the east of the valley is the Andean Cordillera which mainly consists of the Abanico Formation (Cenozoic time period) characterized in the study area by a sequence of andesitic and basaltic lavas with intercalations of pyroclastic and sedimentary rocks, as a undifferentiated conglomerates, sandstones and limonite intercalates (*EMa*) intruded by several stocks, plutons and veins (*Mgh*) of Miocene age. From the Coastal Cordillera into the Argentinian Andes, the Pudahuel ignimbrites (Plip) represent an important deposit attributed to the latest Pleistocene volcanic event and corresponding to a rhyolitic volcanic eruption. The geological record of the Central Depression includes unconsolidated deposits, such as a set of alluvial deposits (*PlHa*), piedmont alluvial deposits (*PlHac*), mass transport deposits (*PlHrm*) and modern fluvial deposits along the current river beds (*Hf*), as well as anthropogenic deposits (*Hra*) (Alfaro et al., 2017; Godoy et al., 2009).

### Methods

The data acquisition in the field was based on indications by Salminem et al. (2005) and the Agricultural and Livestock Service of Chile (Zagal & Sadzawka, 2007). The laboratory analyses were performed in Chile at SERNAGEOMIN.

#### Sampling strategy and preparation for chemical analyses

Urban area samples were taken every 0.5 km^2^, while in peri-urban and rural areas, samples were collected every 1 km^2^ and 5 km^2^, respectively (Reimann et al., 2008). The urban soil samples were taken from open public spaces, such as public playgrounds, wetlands, fields and industrial areas. Three types of soils represent samples from the rural and inter-rural areas: vineyards, fruit trees or corn plantations and sites without plantations or abandoned areas.

In each of the described areas, 2 kg of composite material was sampled. Each sample was formed by five sub-samples: four sub-samples from the corners of a 5 m × 5 m square, and one sub-sample from the center. This method was chosen to ensure a representative and homogeneous sample from the area, considering that the mineralogical composition of soil can vary considerably on the centimeter scale (Salminem et al., 2005).

In order to consider the lateral distribution and vertical mobility of potential pollutants at each point, samples were taken between 20 and 30 cm depth (Alloway, 2013; De Vivo et al., 2018), depending on the soil compaction. To improve data reliability, samples were taken with plastic shovels to avoid contact with any metallic components. Each sample was placed in a polyethylene bag, sealed and labeled. After that, the samples were stored at room temperature at SERNAGEOMIN.

Once the samples arrived in the laboratory, they were dried for a week at 30.1 °C in an electronic oven (Memmert GmbH). Later, the samples were sieved over a screen (2 mm and 0.18 mm mesh size; VWR International). Two fractions were obtained: between 2 mm and 180 µm, and less than 180 µm. From the < 180 µm fraction, 40 g was collected in a polypropylene jar and then ground in an agate rock mortar (ROCKLABS) and ultimately passed through a 200 screen to produce a < 75 µm fraction, which was used for the chemical analysis.

#### Chemical analyses

Thirty-nine major and trace elements from 109 sampling points distributed over the entire study area were analyzed using the X-ray fluorescence (XRF) (Panalytical AXIOS) at SERNAGEOMIN geochemical facility. Analyses include major element oxides (SiO_2_, Al_2_O_3_, TiO_2_, Fe_2_O_3_, CaO, MgO, MnO, Na_2_O, K_2_O and P_2_O_5_) and trace elements (copper [Cu], vanadium [V], chromium [Cr], cobalt [Co], nickel [Ni], zinc [Zn], rubidium [Rb], strontium [Sr], yttrium [Y], zirconium [Zr], niobium [Nb], barium [Ba], lead [Pb], scandium [Sc], arsenic [As], molybdenum [Mo], antimony [Sb], tin [Sn], gold [Au], cadmium [Cd], silver [Ag], bismuth [Bi], tungsten [W], selenium [Se], tellurium [Te] and gallium [Ga]). For quality control, soil calibration standards GRX-2, GRX-5 and GRX-6 (Govindaraju, 1994) were used. Additionally, the instrument was calibrated with rock standards (JA-2 and AGV2) certified by the Geological Survey of Japan (GSJ), United States Geological Survey (USGS) and the Canadian Certified Reference Material Project. These standards were used at the beginning and end of a series of 10 samples.

The C and S analyses were done with the high-temperature combustion method. Measurements were made in a CS cube, model Rapid CS cube, Elementar brand. This method works at a permanent temperature close to 1,200° C, reaching 1,800° C during combustion. In pure oxygen, the contained sulfur is completely converted to SO_2_ with 100% recovery. Carbon is converted to CO_2_ and detected by a true non-dispersive infrared photometer.

The mercury analyses were performed with the MERCUR mercury analyzer kit, Analytikjena, model Mercur Duo Plus Autosample AS 52S. This method is based on the principle of fluorescence, where excited atoms of gaseous mercury absorb radiation from a mercury lamp with a wavelength of 253.7 nm and emit the stored energy in the form of fluorescent light intensity at the same length of wave. The sample is reduced to mercury (0) with a reducing agent (SnCl_2_) and transported by a stream of inert gas, where the separation of the gaseous phase and liquid phase occurs, releasing the gaseous mercury. The separated gas is transported to the fluorescence cell on a gold collector, where the intensity of the emitted light is measured. The analysis of mercury in water, by atomic vapor spectrometry, is based on the EPA 1631 method.

Once results were obtained, a quality control (QC) was performed in accordance with analytical protocols at the SERNAGEOMIN. Quality control mostly involved geochemical variables with concentrations under the detection limit; these variables were replaced by half their value (Reimann et al., 2008) to analyze the matrices of multivariate data. However, measurements with more than 70% results under the detection limit were eliminated; such elimination applied to Au, Cd, Se, Sn and Te analyses.

#### Environmental geochemical baseline map

Following Reimann et al. (2008), the generation of a baseline consists of determining the variation range of the elemental concentrations in the surface environment. In general, the geochemical data do not present a normal distribution; therefore, the calculation of statistical parameters such as the robust coefficient of variation (% CVR) and the interquartile range (IQR) was carried out. The CVR is defined as the ratio between median absolute deviation (MAD) and median (Q_2_), usually expressed in percent. IQR was calculated from the 25^th^ and 75^th^ percentiles (Q_1_ and Q_3_), which contained approximately 50% (median) of the data and determining the geochemical background. Thus, the upper or lower 25 percent of the data are not used in the calculation. From Q_1_ and Q_3_, the upper and lower limits were defined. These correspond to the IQR extended by 1.5 times the length of the box (IQR) toward the maximum and the minimum. This is defined algebraically and the individual values that go beyond these limits are considered extreme or anomalous values (outliers). The study scale, spatial distribution and statistical parameters must be considered when identifying anomalous values (Reimann et al., 2005, 2008) because they could represent unavoidable critical environmental events. Consequently, the identified anomalous values were considered as specific values, which do not represent a data population but likely an important environmental event; therefore, they were not eliminated from the data set.

*Isomeric log-ratio (ilr) transformations* The ilr transformations (Egozcue et al., 2003) permit the transformation of obtained data in weight percent (wt%) and parts per million (ppm) to a dimensionless non-Euclidean space, generally distant from real environmental variations. This transformation is sustained if the obtained data consist of a close data group, where the variables are related to each other and the total of each one equals a constant value (Filzmoser et al., 2009).

The compositional data set consisted of only two components: the measured value for each variable (x) in each sample and the sum of all of the remaining parts of each sample, defined as 1-x. The equation defined for the calculation of *ilr* is:

$$\mathrm{Ilr} \left(\times \right)= \sqrt{\frac{1}{2}\times \mathrm{ln} (\times /1-\times )}$$

One of the most important theoretical properties represented by this transformation is *isometry*, which directly relates Euclidean geometry to the geometric space defined for the transformation (ilr space) such that it is possible to work with the dimensionless transformed variables and, subsequently, relate the results to the original data space.

*Interpolation maps* The graphical representation of the obtained data was made with ArcGIS software (v. 10) by applying the spline line method. This is an interpolation technique that considers the minimum curvature of the sampled surface or, in other words, the sampled area extends and curves into those known points in the sample space, estimating the unknown values. This method is very useful because it permits the inclusion of land structures, such as geological faults and geological unit contacts. It applies to samples with regular spacing, as in this study, although there is a limitation with those samples that have little spreading between one point to another.

#### Pollution source identification

Principal component analysis (PCA) and factorial analysis (FA) are one of the most applied and important multivariate statistical methods (Filzmoser et al., 2018). Factor analysis (FA) was used to identify different sources of pollution in the study area. The FA allows to explain the correlation pattern of a set of observed data through the identification of principal components. For a better interpretation, these principal components are rotated and are used such that the variables load predominantly onto one component. The direction of each principal component is expressed by its loadings which convey the relation to the original variables. The interpretation of the factors is made based on the loads. Therefore, each factor demonstrates different elemental correlations that are indicative of specific sources (Reimann et al., 2008). The plot of factor loadings in a graph shows by its x-axis the relative amount of variability by each single factor in the FA model. The y-axis is scaled from + 1 through 0 to -1 and shows the factor loadings of the different variables entering each factor. Dashed lines at values of + 0.5 and –0.5 help to distinguish the important (> + 0.5) from the less important variables (< – 0.5).

In this study, all values between ≥ 0.5 and 1 are considered as the main source, while values between 0 y < 0.5 are considered as secondary sources. This study identified eleven principal factors, which represent eleven main pollutant sources that explain 84% of the total variance.

Prior to FA, the elemental composition was calculated from the oxides following the formula:

$$x \left(\mathrm{ppm}\right) =(\mathrm{AW}x /\mathrm{MW}x)\times X(wt\%)\times 10.000$$

where *x*: Elemental composition proportional to oxide, AWx: Atomic weight of element, MWx: Molecular weight of element, *X* (wt%): Concentration in wt% of oxide x.

## Results and discussion

### Geochemical baseline

**Fig. 2:**
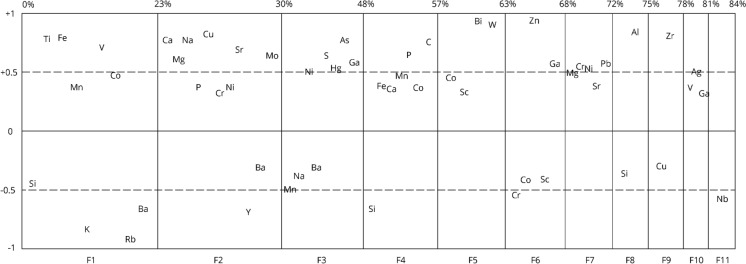
Result obtained according to factor analysis. In the table, 11 factors are observed corresponding to the different elementary associations, selected according to the loading obtained

The results of major and trace elements and statistical parameters are presented in Tables 1 and 2, respectively. Data allow characterization of variations in elemental concentrations in the study area (i.e., the baseline). This information is presented in terms of provenance and distribution of each variable association obtained from FA (Fig. 2).

**Table 1:** Presentation of major elements concentrations obtained from trace elements in analyzed soils. Element: elements analyzed, wt%: weight percentage, n: 109 samples analyzed, Sum of major oxides: total sum in percentage by weight of each oxide, Minimum: smallest values in wt%, Q_1_: 1st quartile (25%), Median (50%), Q_3_: 3rd quartile (75%), Upper limit: max {*x*, [*x* ≤ Q_3_ + 1.5 * (*Q*_*3*_–*Q*_*1*_)]}, Maximum: highest values, CVR: Robust Coefficient of Variation (percent)

Oxides	Unit	n	Sum of major oxides (wt%)	Minimum	Q_1_	Median	Q_3_	Upper limit	Maximum	% CVR
SiO_2_	wt%	109	55.4	48.1	54.6	55.2	56.0	57.9	68.4	2.0
Al_2_O_3_	wt%	109	16.6	14.4	16.2	16.6	17.0	18.1	21.4	3.5
TiO_2_	wt%	109	1.0	0.4	0.9	1.0	1.0	1.2	1.6	8.9
Fe_2_O_3_	wt%	109	8.2	3.1	8.0	8.3	8.7	9.2	10.8	6.2
CaO	wt%	109	3.4	1.1	2.9	3.2	3.9	5.8	6.8	20.3
MgO	wt%	109	3.3	0.5	3.0	3.5	3.7	3.7	5.2	15.8
MnO	wt%	109	0.2	0.1	0.1	0.2	0.2	0.2	0.3	9.9
Na_2_O	wt%	109	2.2	1.3	1.9	2.2	2.6	2.6	2.9	24.3
K_2_O	wt%	109	1.9	0.6	1.8	1.9	2.0	2.3	3.0	8.6
P_2_O_5_	wt%	109	0.3	0.1	0.3	0.3	0.3	0.5	0.8	18.8

**Table 2:** Presentation of the concentrations in ppm and wt% for C and S. Element: elements analyzed, wt%: weight percentage, n: 109 samples analyzed, Minimum: smallest values in wt%, Q_1_: 1st quartile (25%), Median (50%), Q_3_: 3rd quartile (75%), Upper limit: max {*x*, [*x* ≤ Q_3_ + 1.5 * (*Q*_*3*_–*Q*_*1*_)]}, Maximum: highest values, CVR: Robust Coefficient of Variation (percent). < 10: Value below the detection limit (10.0 ppm), ND: not determined

Trace elements									
Elements	Unit	n	Minimum	Q_1_	Median	Q_3_	Upper limit	Maximum	% CVR
Cu	ppm	109	34.8	226.0	420.0	677.3	677.3	2497.1	72.1
V	ppm	109	60.4	163.6	183.0	195.0	195.0	227.2	14.1
Cr	ppm	109	5.0	25.5	30.3	34.7	47.8	206.6	21.6
Co	ppm	109	7.7	25.2	26.6	29.8	38.1	46.5	9.6
Ni	ppm	109	2.5	10.7	14.2	17.5	17.5	26.4	35.8
Zn	ppm	109	12.0	83.5	92.2	104.0	140.0	180.3	17.3
Rb	ppm	109	27.9	63.1	69.4	75.5	93.5	146.4	13.4
Sr	ppm	109	80.1	374.0	406.6	455.1	455.1	543.3	13.6
Y	ppm	109	19.0	21.1	22.1	23.4	26.0	27.4	7.4
Zr	ppm	109	118.6	158.1	166.8	175.9	204.6	332.9	8.3
Nb	ppm	109	2.5	2.5	2.5	6.0	12.7	12.7	ND
Ba	ppm	109	271.5	348.0	374.1	405.7	470.0	751.5	10.6
Pb	ppm	109	2.5	14.0	21.3	27.7	27.7	61.7	49.2
Sc	ppm	109	6.3	16.3	20.3	25.5	40.0	110.1	35.1
C	wt%	109	0.2	1.2	1.5	1.8	3.1	5.6	27.3
S	wt%	109	0.02	0.1	0.1	0.3	0.3	0.9	97.2
Hg	ppm	109	0.01	0.02	0.1	0.1	0.1	0.2	ND
As	ppm	109	10.0	10.0	10.0	20.0	55.5	134.6	ND
Mo	ppm	109	2.5	2.5	5.0	5.8	20.3	26.5	ND
Sb	ppm	109	5.0	5.0	12.6	25.6	25.6	79.4	ND
Ag	ppm	109	0.5	0.5	1.0	1.7	1.7	7.6	ND
Bi	ppm	109	5.0	5.0	5.0	21.6	21.6	33.0	ND
W	ppm	109	5.0	5.0	11.0	25.4	25.4	59.4	ND
Ga	ppm	109	0.5	12.2	79.3	108.7	108.7	142.0	114.0

#### Continental crustal origin

The concentrations of Fe_2_O_3_ vary between 3.1 and 10.8 wt% and for TiO_2_ between 0.4 and 1.6 wt%. In both cases, highest values are observed in the eastern sector, mainly on rural soils of the study area, consistent with an andesitic-basaltic lithology of the Abanico Formation (*EMa*) that is known for its mafic mineral content. SiO_2_, K_2_O, Rb and Ba show highest concentrations (68.4 w%, 3.0 wt%, 146.4 ppm and 751.5 ppm, respectively) in alluvial and colluvial deposits (*PlHac*) close to the Las Chilcas Formation (*Kilc*) outcrops at the western edge of the study area, and they are showing an association with rhyolitic ignimbrite deposits of Pudahuel (*Plip*) in the Machalí area, corresponding mostly located in rural areas. In general, the concentrations of the major oxides are within the compositional ranges observed in the Abanico Formation by Vergara et al. (2004) (SiO_2_: 49.94–58.54 wt%, Al_2_O_3_: 15.53–16.97 wt%, TiO_2_: 0.73–1.55 wt%, Fe_2_O_3_: 8.25–13 wt%, CaO: 2.91–3.92 wt%, MgO: 3.59–3.80 wt%, MnO: 0.13–0.19 wt%, Na_2_O: 3.07–3.55 wt%, K_2_O: 0.54–2.04 wt% and P_2_O_5_: 0.27–0.35 wt%). Therefore, the origin of these elements has been considered mostly to a lithologic origin.

This pattern is consistent with the first identified source (FA, factor 1, Fig. 2) and is subdivided into two primary sources: one from mafic minerals characterized by an elemental association of Ti, Fe, V, Mn and Co (Vergara et al., 2004) with ≥ 0.5 loading, approximately, and another of intermediate to acid rock with K, Rb, Ba and Si (Oyarzún et al., 2016) with ≥ 0.5 loading.

The concentrations of C and P_2_O_5_ vary between 0.2–5.6 wt% and 0.1–0.8%, respectively. The highest concentrations of both elements predominate in the urban soils of the city of Rancagua and the town of Graneros, as well as in the peri-urban soils, located between both localities and used for agriculture crops. The elemental association is given by the higher loading (≥ 0.5) of the fourth factor (Fig. 2), which is consistent with the presence of C as an important reservoir in agricultural soils and in the terrestrial biosphere (Kabata-Pendias, 2011). This is supported by the content of organic matter (4%) in soils of the study area (Bonilla & Johnson, 2012), as well as the random distribution in any study area.

Also, the decomposition of organic matter and crop residues contribute to P availability (Noack et al., 2012). It is possible to detect P in organic and inorganic forms, and in general, inorganic P is associated with Fe and Mn in soils with acid pH and, with Ca in alkaline soils (Arenberg & Arai, 2018). Kabata-Pendias (2011) explained that there is a low occurrence of crystalline forms of phosphate minerals in soils, despite the role that metastable phosphates play in pedogenesis.

Another association that permits the traceability of the parent rock is given by factor 6 (Fig. 2), where the elements with major loading (≥ 0.5) are Zn and Ga (maximum values of 180.3 ppm and 142.0 ppm, respectively). The higher Ga concentrations are in the north of the study area in rural and peri-urban soils and associated with clay-rich alluvial deposits, which is consistent with Ga correlating with the clay fraction (Kabata-Pendias, 2011).

#### Activities related to Cu extraction

**Fig. 3:**
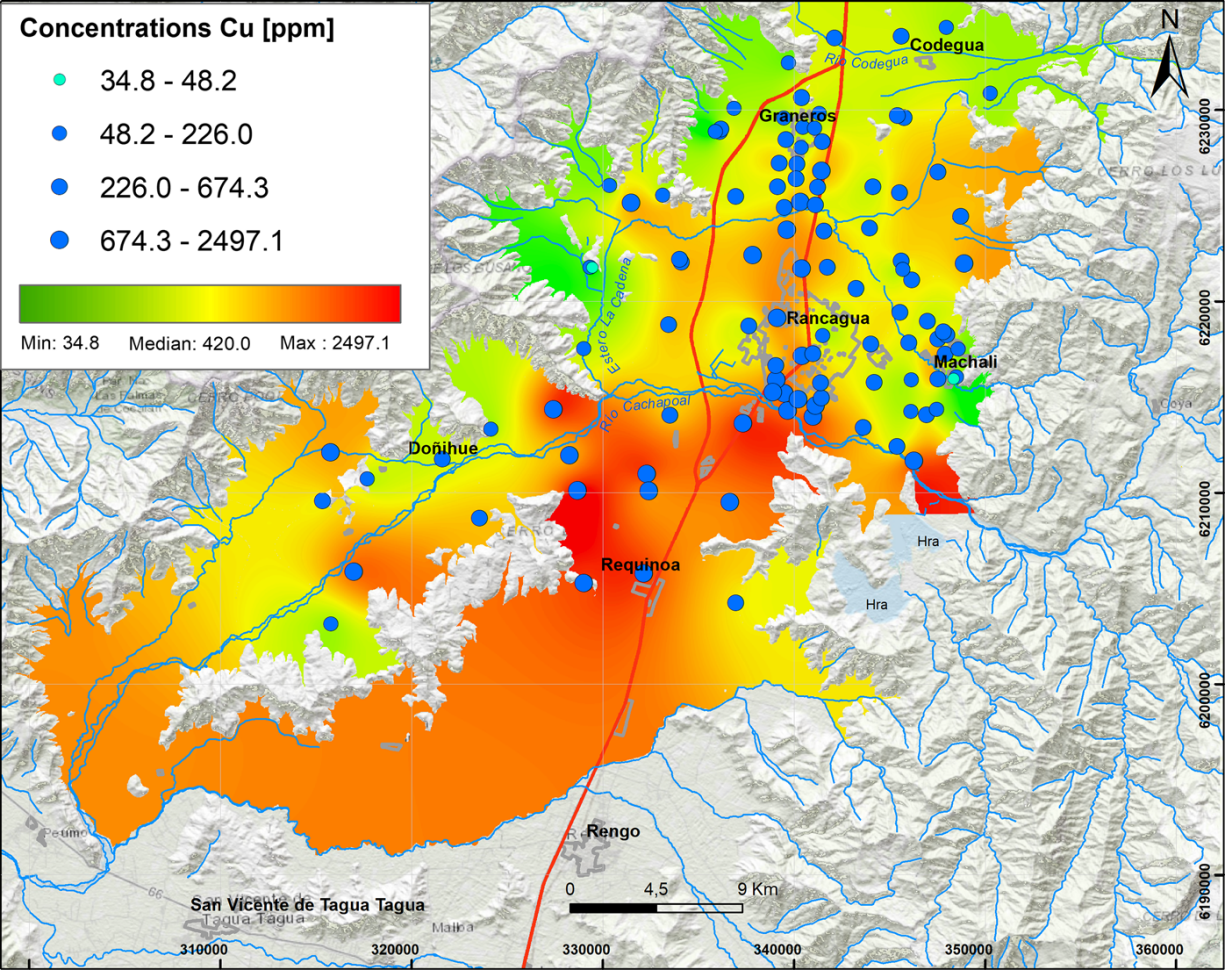
Elemental distribution of copper with major concentrations south of the study area. In the upper left box, the results of 109 samples analyzed are indicated, whose concentrations vary from the minimum value (34.8 ppm) to the maximum value (2497.1 ppm), with Q_1_: 226.0 ppm and Q_3_: 677.3 ppm

*Irrigation water* Copper exhibits high concentrations (226.0–677.3 ppm) with a maximum of 2500 ppm approximately (Table 2, Fig. 3). Highest concentrations of > 1000 ppm are found along the southern border of the Cachapoal River (Fig. 3) in mainly rural soils. This area also shows a median of Mo concentrations of 5.0 ppm and maximum of 26.5 ppm, which suggests Mo can trace various activities related to Cu extraction, such as the use of water from the Cachapoal River for irrigation of agricola soils (Sudzuki, 1960). Upstream areas would be enriched in Cu, both in the soluble and sediment fractions because of constant input from the Barahona 1 and 2 tailing deposits (Kelm et al., 2009). Previous studies (Cade-Idepe, 2004) indicate that metal and metalloid concentrations in waters of the Cachapoal River such as Cu (0.2–810 mg/L), Mo (0.010–0.020 mg/L), Zn (0.04–0.22 mg/L) and As (0.010–0.140 mg/L), among others, mostly exceed the limits established in the Chilean irrigation water standard (Nch 1,333/78; 0.20 mg/L; 0.010 mg/L; 2 mg/L and 0.10 mg/L, respectively). Geochemical analyses from sediments from the same river indicate concentrations of Cu (200–1400 ppm), Mo (20–140 ppm), As (20–50 ppm), Sb (10–22 ppm) and B (40–140 ppm) (Lacassie, 2008). These increased concentrations are consistent with the values observed in the present study.

The second elemental association identified in factor 2 by Cu, Na, Ca, Sr, Mo, Mg (≥ 0.5) and P, Cr and Ni (0.5 ≤) shows a complex signal of mixed origin (Fig. 2). The association of these elements with Ca, Mg, Sr and Na indicate an alluvial type deposit (*PlHa*_*1*_* [a]*) located south of Rancagua, that suggest ancient pathways of the Cachapoal River. The origin of Ca, Mg and Sr elements could be due to carbonate rocks of Abanico and Farelllones formations, that were dissolved in the Chachapoal river.

**Fig. 4:**
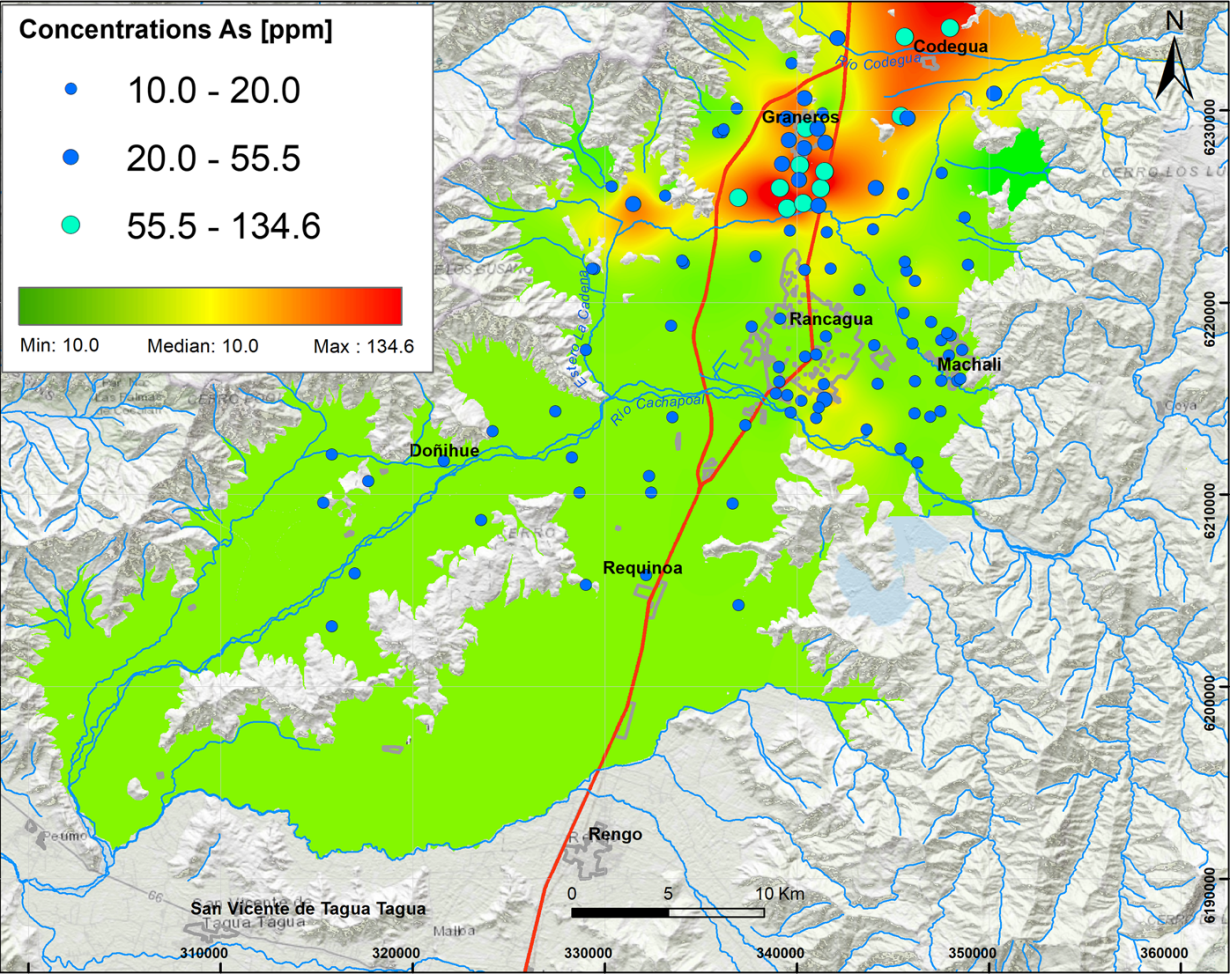
Elemental distribution of arsenic in the study area. In the upper left box, the results of 109 samples analyzed are indicated, whose concentrations vary from the minimum value (10 ppm) to the maximum value (134.6 ppm), with Q_1_: 10.0 ppm and Q_3_: 20.0 ppm

*Emissions of particulate material* Highest concentrations of As are found in the northern study area, mainly in peri-urban and rural soils for agricultural use (axis Graneros–Codegua to the Tuniche area) (Fig. 4). Here, the concentrations vary between 10.0 and 134.6 ppm. These values are compatible with high concentrations of As in Graneros soils (333 mg/kg), which are associated with the particulate material emitted from the Caletones smelter, which is consequently deposited in agricultural soils (Richter et al., 2004). Also, in the La Leonera sector, Romo-Kroger et al. (1994) detected high levels of As in the fine particulate material emitted by the Caletones smelter, related to Zn and S emissions.

**Fig. 5:**
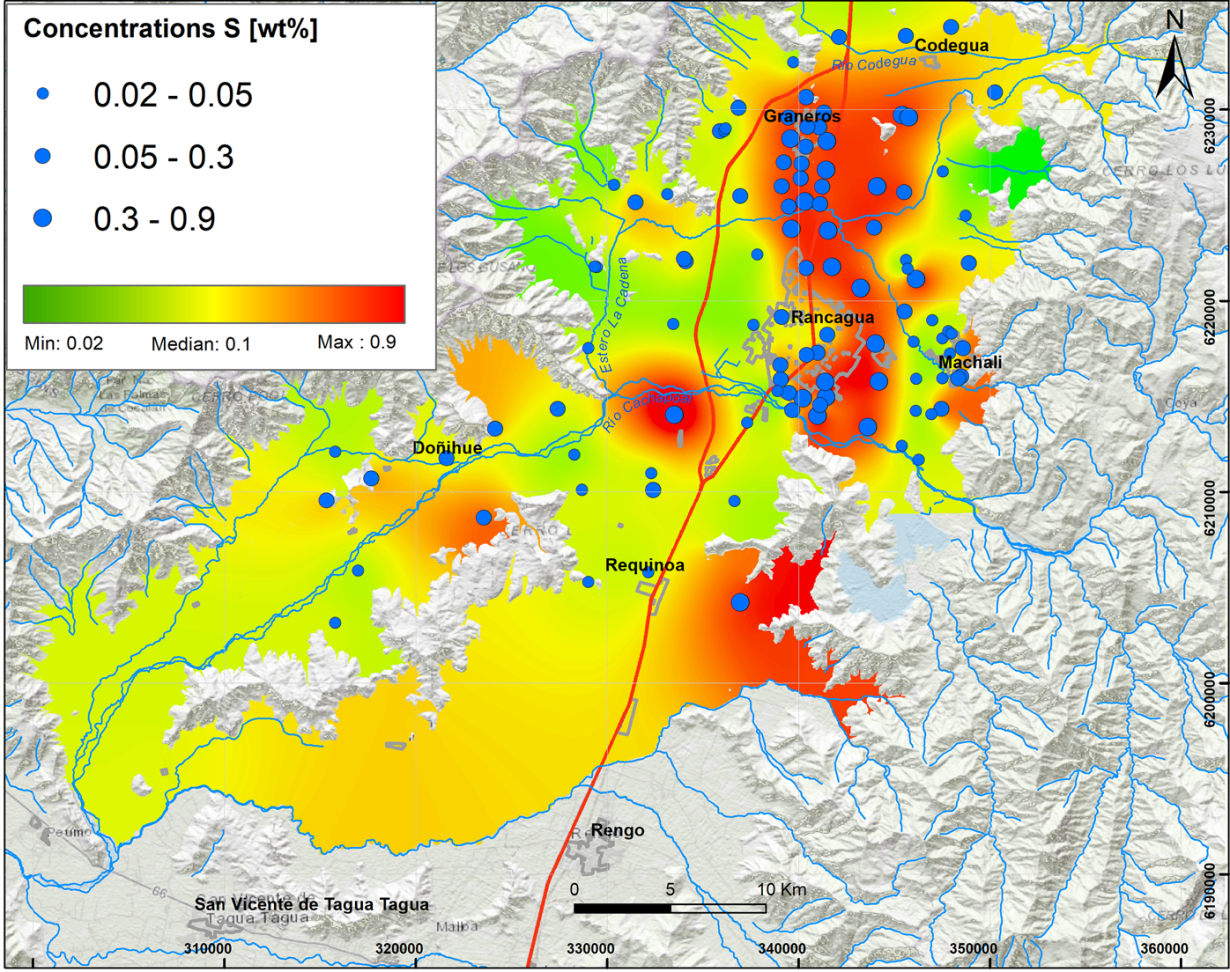
Elemental distribution of sulfur in the study area. In the upper left box, the results of 109 samples analyzed are indicated, whose concentrations vary from the minimum value (0.02 wt%) to the maximum value (0.9 wt%), with Q_1_: 0.1 wt% and Q_3_: 0.3 wt%

The median sulfur concentrations in soils vary between 0.01 and 0.3 wt% with a maximum of 0.9 wt%. High concentrations in soils of S are present along the Graneros–Rancagua axis (Fig. 5), in urban and peri-urban soils and to a lesser extent in rural soils. The median concentrations of Hg vary between 0.02 ppm and 0.1 ppm (maximum of 0.2 ppm) in the study area. Hg presents a distribution marked with high concentrations in the central and eastern areas, in the three types of soils of the study zone, and it is related to the use of pesticides in the area.

Average Ni concentrations vary between 10.7 ppm and 17.5 ppm with a maximum of 26.4 ppm. These values are mainly observed in the soils of Rancagua and Graneros, as well as in the surrounding soils. The spatial relationship between all these variables is consistent with the third elemental association (≥ 0.5) observed in factor 3 (As, S, Ga, Hg, Ni) (Fig. 2).

Arsenic, Ga and Hg tend to interact with S and Ni which is seen in the Earth’s crust (Kabata-Pendias, 2011), but the origin of all these elements can be related to emissions from the Caletones smelter, where an association of As and S already has been observed (Romo Kroger, 1994). In 1994, Caletones smelter was declared as a saturated zone for sulfur dioxide (SO_2_) and particulate material (Decreto179, 1994). Therefore, high concentrations of S can be explained by the deposition of atmospheric particulate material from the Caletones smelter (Gallardo et al., 2002), as well as by soil fertilization processes that use copper sulfate (Ahumada et al., 2004). Highest concentrations of Hg are related to soils with higher organic C content and can be linked to the combustion of fossil fuel and pesticide use (Ramírez y Lacasaña, 2001). The prevailing wind pattern from Los Andes Mountains to the valley of the O´Higgins region could thus be explaining the transport of these elements from the Caletones smelter into Central Depression (Sanhueza, 2006).

#### Metal alloys

The source of metal alloys was traced by the elementary association of Bi (33.0 ppm) and W (59.4 ppm), determined by the major loading (≥ 0.5) of factor 5. The chemical composition of upper continental crust indicates for Bi and W, concentrations of 127 and 2 ppm, respectively (Taylor and McLenan, 2009). However, usually, Bi is extracted as a by-product from exploitation of Cu, Ag, Au and W ore and is mostly used for low melting point alloys. In contrast, W is used for high hardness alloys that need to have a high wear out resistance (Kabata-Pendias, 2011). One of the main alloys to use W is called cemented tungsten carbide or “hard metal” with 5% to 10% Co that acts as a particle binder (ITIA, 2011). The relationship between these three elements is associated with the use of machinery, mechanical parts and mining and agricultural equipment, even though a natural origin cannot be discarded.

This main signal is complicated by a secondary signal of Co (7.7–46.5 ppm) and Sc (6.3–110.1 ppm), determined by its lower loading (< 0.5) in the same factor 5. The highest concentrations were found in the southern part, in peri-urban and rural soils around the city of Rancagua in areas with high Fe_2_O_3_ and TiO_2_. Therefore, these oxides control the Co content in soils, which would explain the Co affinity with Sc (Kabata-Pendias, 2011). These values are higher than those (Co: 4–29 ppm; Sc: 5–28 ppm) determined in the volcanic series at 33.6°S by Muñoz et al. (2020).

#### Oil combustion

**Fig. 6:**
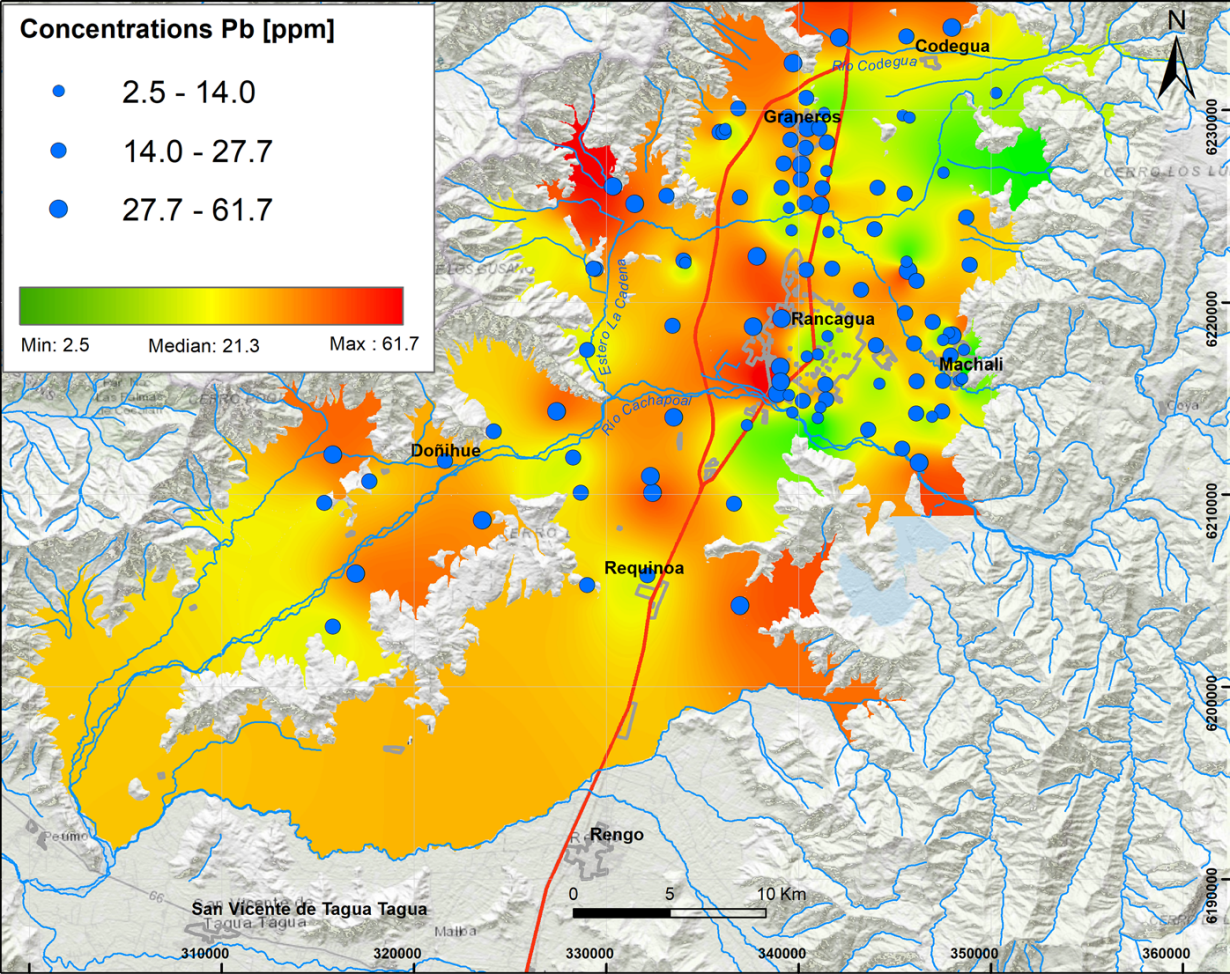
Elemental distribution of lead in the study area. In the upper left box, the results of 109 samples analyzed are indicated, whose concentrations vary from the minimum value (2.5 ppm) to the maximum value (61.7 ppm), with Q_1_: 13.0 ppm and Q_3_: 27.7 ppm

**Fig. 7:**
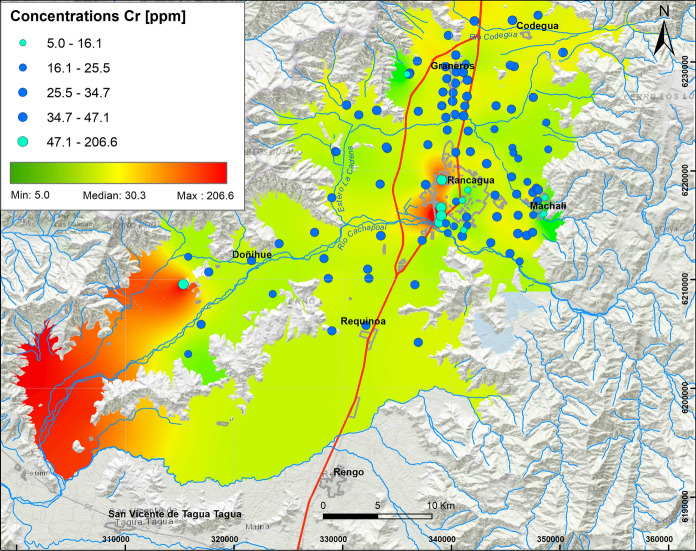
Elemental distribution of Cr, whit higher concentrations in soils of Rancagua City. In the upper left box, the results of 109 samples analyzed are indicated, whose concentrations vary from the minimum value (5 ppm) to the maximum value (206.6 ppm), with Q_1_: 25.5 ppm, Q_3_: 34.7 ppm

**Fig. 8:**
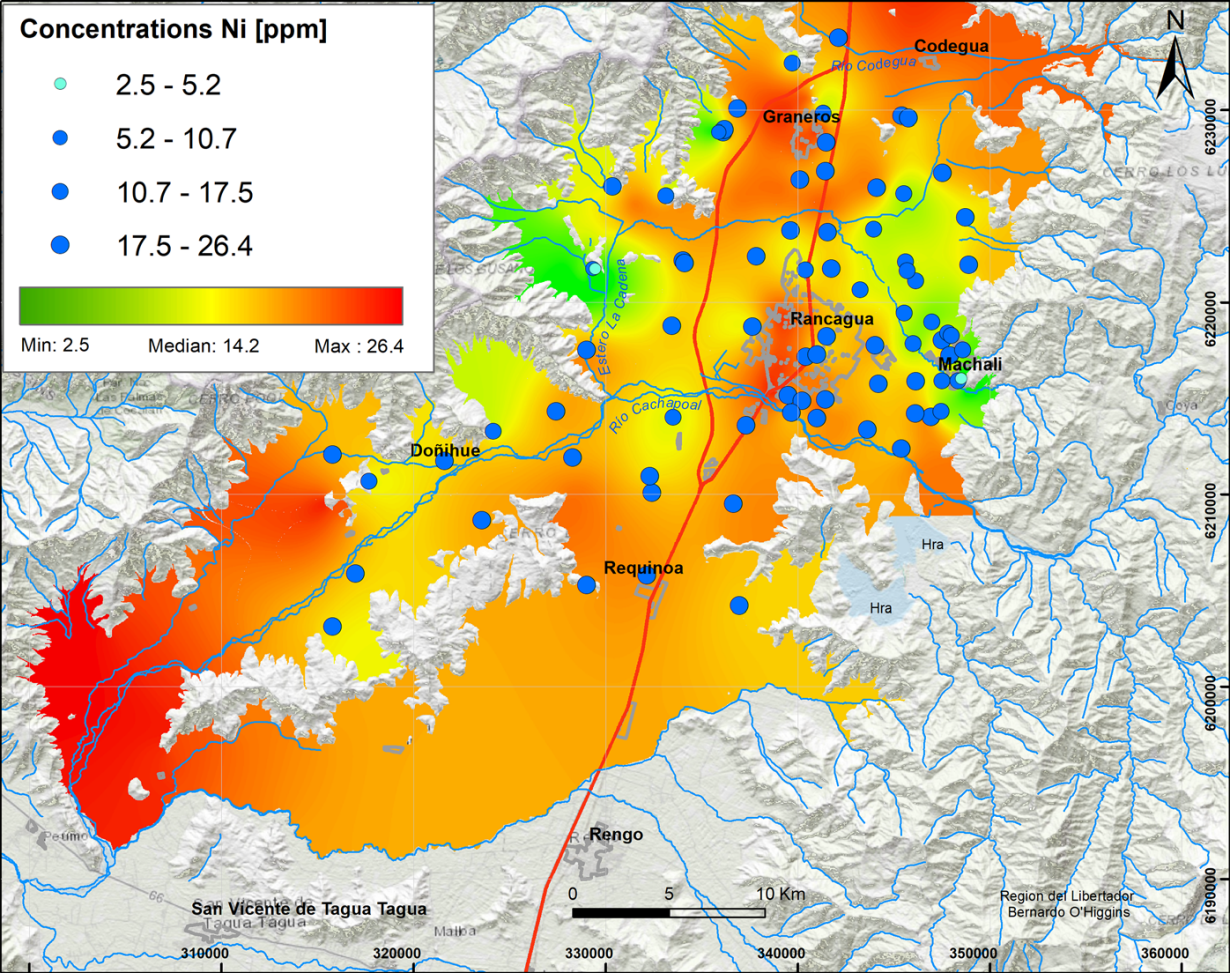
Elemental distribution of Ni with major concentration around of Rancagua and Graneros. In the upper left box, the results of 109 samples analyzed are indicated, whose concentrations vary from the minimum value (2.5 ppm) to the maximum value (26.4 ppm), with Q_1_: 10.7 ppm and Q_3_: 17.5 ppm

According to the highest concentrations and association between Pb (61.7 ppm, Fig. 6), Cr (206.6 ppm, Fig. 7) and Ni (26.4 ppm, Fig. 8) in factor 7, these elements are mostly concentrated in soils of urban areas, specifically on the western side of Rancagua city, where the Cr concentrations were particularly high (> 100 ppm). Cr values exceed the norms established in some countries as Canada, Germany and Brazil, among others.

These values are higher than Pb (5–21 ppm) and Cr (20–90 ppm) concentrations described in volcanic series at 33.6° S (Muñoz et al., 2020). According to the results, Pb could be related to industrial activity (Salminem et al., 2005). Cr has been associated with traffic (Salma & Maenhaut, 2005), while Valdés et al. (2013) recognized Cr and Ni as oil combustion tracers.

### Comparison between elemental concentrations and international standards

The results obtained were compared with international standards in accordance with the provisions of Chilean law (Article 11 Law 19.300). For the purposes of this study, the norm of Canada, Netherlands, Germany and Brazil (Sao Paulo) has been considered due to the comparison of variables (use of soils and chemical elements) that can be established between the country study and the selected countries (Table 3).

**Table 3:** Canada, Netherlands, Germany and Brazil international standards for dry soils for agricultural use

International Standards				
Countries	Canada	Netherlands	Germany	Brazil
Dry soil (mg/Kg)				
Use	Agriculture	^1^VI	–	Agriculture
Elements				
As	12	55	20	35
Co	35	240	–	35
Cr^Total^	64	380	100	150
Cu	63	190	100	200
Hg	6.6	10	1	12
Mo	15	200	–	50
Ni	50	210	70	70
Pb	70	530	100	180
Sb	20	15	–	–
V	130	250	–	–
Zn	200	720	200	450

^1^Intervention Value: level above which the functional properties of the soil for humans, plants and animals would be seriously deteriorated

Both Canada and Brazil establish norms according to the use of soils (agricultural and residential), while Netherlands defines limits between a satisfactory recovery of soils and deteriorated soils. Germany delimits the metal content according to the types of soils (clay, loamy and sandy). For the purposes of this study, the highest values of any of them have been considered.

#### Concentrations of Cu, Mo and Zn

The Cu reach values up to 2500 ppm (Fig. 3), meaning that 75% of the samples are above international norm for agricultural soils, such as in Canada (63 ppm), Holland (190 ppm), Germany (100 ppm) and Brazil (200 ppm). They also exceed the proposed values for the sewage sludge of soils (100 ppm) and toxic tolerable content (200 ppm) in the European Community (Galán & Romero, 2008). Ahumada (2004) obtained soluble Cu data from soils of south of the Cachapoal River with values that varied between 125 and 200 ppm and close to Graneros where values varied between 63 and 70 ppm. This spatial distribution is consistent with the enrichment in observed Cu from previous studies of irrigation water (Richter et al., 2004). This enrichment is most probably due to the contribution of Cu from the tailings of Barahona 1 and Barahona 2, as well as fertilization of soil with copper sulfate.

Eight percent of the highest Mo (26.5 ppm) concentrations exceed the Canadian norm (5 ppm). In contrast, Zn does to do values (180.3 ppm) do not exceed any international norm for agricultural soils.

#### Concentrations of As, S, Ga, Hg and Ni

Twenty-one percent of the highest total concentrations of As in the present study area exceed the norm for dry soils of agricultural use in Canada (12 ppm), Holland (55 ppm), Germany (20 ppm) and Brazil (35 ppm). The As–S association could be related to fine (MP_2.5_) and coarser (MP_10_) particulate emissions from the Caletones smelter (Romo-Kroger, 1994). In 1994, Caletones was declared as saturated sulfur anhydride (SO_2_) area (Decreto179, 1994), and this explains why a decontamination plan was implemented in 1998 (Decreto81, 1998), which led to a decrease in particulate concentrations. Currently, the norm considers maximum emission limits (47.680 ton/year; ≤ 600 ppm daily) of sulfur (SO_2_), As (130 ton/year; ≤ 1 mg/Nm^3^ month) and Hg (≤ 0,1 mg/Nm^3^). It is known that these emissions also contain other metals, such as Pb and Ni (Kavouras et al., 2001) and, in addition, that pesticides used in agriculture contain Hg (Ramírez & Lacasaña, 2001).

#### Concentrations of Pb, Cr, Ni, Hg, Zn and Mn

The association of Pb, Cr and Ni is mostly concentrated in urban areas, more specifically on the west side of Rancagua city where Cr concentrations are particularly high (> 100 ppm). The highest Cr values represent 3% of the total and are mostly located in Machalí, Codegua and Rancagua urban soils. These exceed the Canadian (64 ppm), German (100 ppm) and Brazilian (150 ppm) regulations. Chromium has also been associated with vehicular traffic (Salma & Maenhaut, 2005), as well as it is an oil combustion tracer along with Ni (Valdés et al., 2013). In general, neither Pb, Hg, Ni nor Zn exceeded critical values defined in the contrasting regulations.

## Conclusions

Major soil elemental associations identified in this study around Rancagua city in the years 2012 and 2013 show that they are generally a mix of natural and anthropic sources. The results show that the distribution of oxides and some trace elements (Rb, Ba, Zn, Ga) V, Sr is controlled by lithology, whereas Cu, Mo, As, S, Hg, Ni, Bi, W, Pb and Cr are controlled mainly by anthropogenic sources. Most of these results were above the international regulations for agricultural soils (e.g., Canada, Holland, Germany and Brazil) and represent a serious risk to the environment and human health. The distribution and concentrations of Cu and Mo are mainly due to transportation by the fluvial system related to tailings from mining activities. Molybdenum shows a similar distribution as Cu; thus, the same origin is suggested. The highest As values are observed in the northern part of the study area in peri-urban and rural soils, specifically on the Graneros–Codegua axis, where at least 21% of the highest concentrations are higher than the German, Brazilian and Canadian regulations. These concentrations are related to the atmospheric deposition of material emitted by the Caletones smelter. The highest concentrations of Cr occur in the urban soils of Rancagua and Machalí. The origin of Cr is related to a natural sources and oil combustion. The maximum concentration of V is close to 227.2 ppm; V most probably has the same origin as Cr. The highest values of Pb are distributed at the western limit of the Central Depression and associated with outcrops of the Las Chilcas Formation and also in the Rancagua city area. Average Zn vary between 83.5 and 104.0 ppm, with a maximum of 180.3 ppm; the concentrations are attributed to emissions related to mining activity. Finally, Co, as another environmentally harmful element, indicates average values between 25.2 and 39.8 ppm, with a maximum close to 46.5 ppm. The origin of this element is related to the parent rock, in situ lithology. It is suggested that major variations in elemental concentrations (Cu, Mo, As, S, Hg, Ni, Bi, W, Pb and Cr) are mainly a result of anthropogenic sources rather than natural contributions. Anthropogenic sources produce contaminations that are a factor of 10 higher than established international regulations. Ultimately, this work is a contribution to show the necessity to reduce soil pollution to protect human health in urban, sub-urban and rural areas close to mining activities.

## Supplementary Information

Below is the link to the electronic supplementary material.

Supplementary file1 (XLSX 2249 kb)

Supplementary file1 (XLSX 2249 kb)

Supplementary file1 (XLSX 2249 kb)

Supplementary file1 (XLSX 2249 kb)

Supplementary file1 (XLSX 2249 kb)

Supplementary file1 (XLSX 2249 kb)

Supplementary file1 (XLSX 2249 kb)

Supplementary file1 (XLSX 2249 kb)

Supplementary file1 (XLSX 2249 kb)

Supplementary file1 (XLSX 2249 kb)

Supplementary file1 (XLSX 2249 kb)

Supplementary file1 (XLSX 2249 kb)

Supplementary file1 (XLSX 2249 kb)

Supplementary file1 (XLSX 2249 kb)

Supplementary file1 (XLSX 2249 kb)

Supplementary file1 (XLSX 2249 kb)

Supplementary file1 (XLSX 2249 kb)

Supplementary file1 (XLSX 2249 kb)

Supplementary file1 (XLSX 2249 kb)

Supplementary file1 (XLSX 2249 kb)

Supplementary file1 (XLSX 2249 kb)

Supplementary file1 (XLSX 2249 kb)

Supplementary file1 (XLSX 2249 kb)

Supplementary file1 (XLSX 2249 kb)

Supplementary file1 (XLSX 2249 kb)

Supplementary file1 (XLSX 2249 kb)

Supplementary file1 (XLSX 2249 kb)

Supplementary file1 (XLSX 2249 kb)

Supplementary file1 (XLSX 2249 kb)

Supplementary file1 (XLSX 2249 kb)

Supplementary file1 (XLSX 2249 kb)

Supplementary file1 (XLSX 2249 kb)

Supplementary file1 (XLSX 2249 kb)

Supplementary file1 (XLSX 2249 kb)

Supplementary file1 (XLSX 2249 kb)

Supplementary file1 (XLSX 2249 kb)

Supplementary file1 (XLSX 2249 kb)

Supplementary file1 (XLSX 2249 kb)

Supplementary file1 (XLSX 2249 kb)

Supplementary file1 (XLSX 2249 kb)

Supplementary file1 (XLSX 2249 kb)

Supplementary file1 (XLSX 2249 kb)

Supplementary file1 (XLSX 2249 kb)

## Data Availability

Original database is attached.
